# Supplementary material for: Assessment of risk to hoary squash bees (Peponapis pruinosa) and other ground-nesting bees from systemic insecticides in agricultural soil
Source: Sci Rep. 2019 Aug 14;9:11870. doi: 10.1038/s41598-019-47805-1 (PMC6694176; doi:10.1038/s41598-019-47805-1)
Supplement: Supplementary file 1 — Electronic Supplementary Information [file 41598_2019_47805_MOESM1_ESM.docx]

**Assessment of risk to hoary squash bees (*Peponapis pruinosa*) and other ground-nesting bees from systemic insecticides in agricultural soil**

**Electronic Supplementary Information**

D. Susan Willis Chan^1^, Ryan S. Prosser^1^, Jose L. Rodríguez-Gil^2^, Nigel E. Raine^1^

^1^ School of Environmental Sciences, University of Guelph, Guelph, Ontario, N1G 2W1, Canada

^2^ Department of Biology, University of Ottawa, Ottawa, Ontario, K1N 6N5, Canada

Email: [dchan05@uoguelph.ca](mailto:dchan05@uoguelph.ca) [nraine@uoguelph.ca](mailto:nraine@uoguelph.ca)

**Figure S1**. Environmental Exposure Distribution (EED) for acute exposure to imidacloprid in soil from field crops (corn, soybeans, wheat) based on a government dataset^1^. Soil samples were taken from 0-15 cm depth in southern Ontario, 2016. Effects benchmark concentrations are for solitary ground-nesting bees based on the hoary squash bee (*Peponapis pruinosa*) acute exposure amounts (48 h, 2.23 g soil). Effect benchmarks (i.e. honey bee geometric mean LC_50_ = solid great vertical line, honey bee lowest LC_50_ = dotted grey vertical line, and the solitary bee surrogate LC_50_ = grey dashed vertical line) are represented by vertical lines on the EED. Exceedance of these endpoints is calculated by subtracting the cumulative probability from one. Grey horizontal lines represent individual soil samples below the analytical limit of detection, and black dots indicate samples for which insecticide residues could be quantified. The red line indicates the line of best fit to the data via maximum likelihood estimation (MLE) with associated 95% confidence intervals (pink shading).


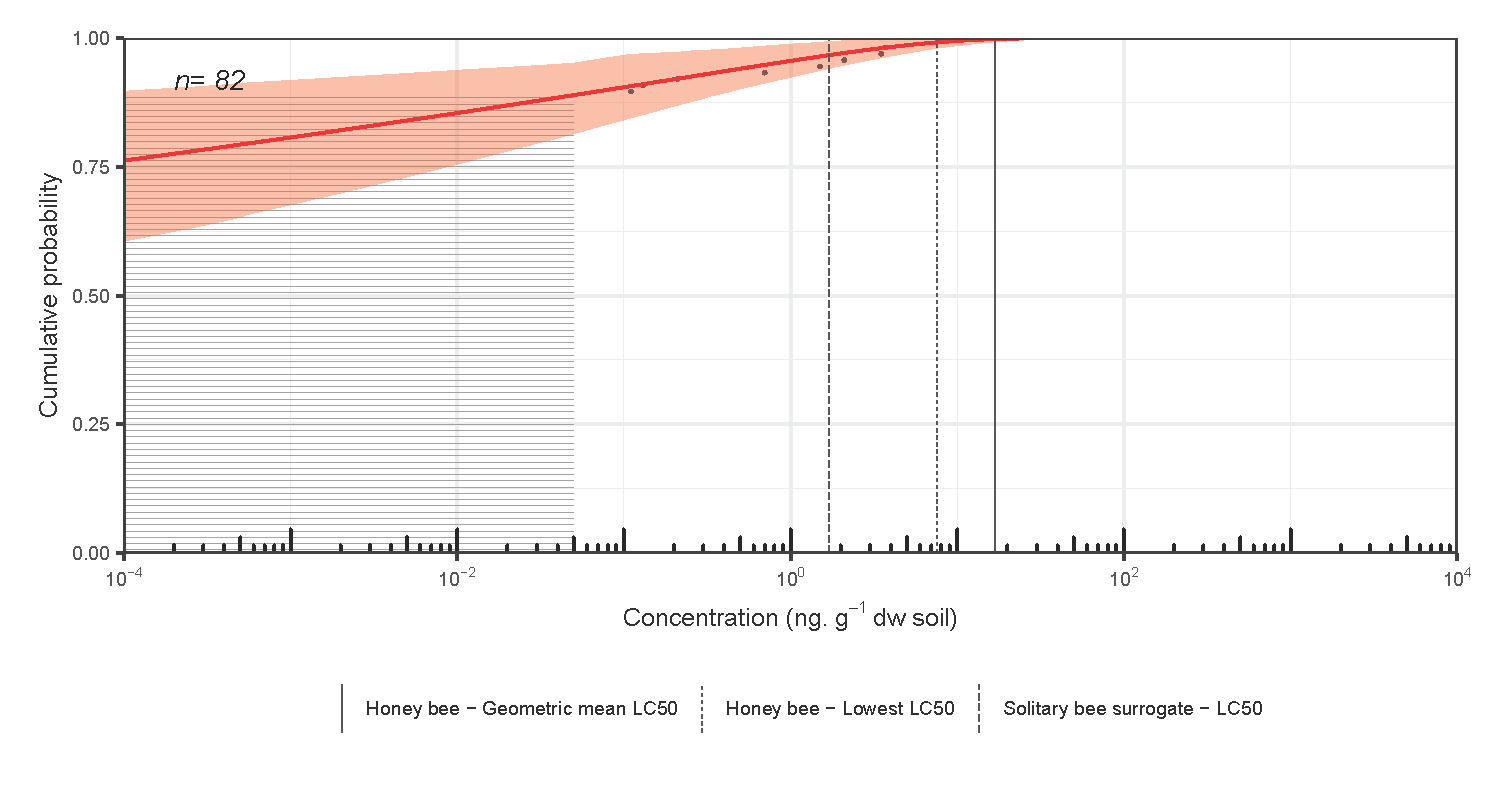


**Figure S2**. Environmental Exposure Distribution (EED) for chronic exposure to imidacloprid in soil from field crops (corn, soybeans, wheat) based on a government dataset^1^. Soil samples were taken from 0-15 cm depth in southern Ontario, 2016. Effects benchmark concentrations are for solitary ground-nesting bees based on the hoary squash bee (*Peponapis pruinosa*) acute exposure amounts (30 days, 33.5 g soil). Effect benchmarks (i.e. honey bee geometric mean LC_50_ = solid great vertical line, honey bee lowest LC_50_ = dotted grey vertical line, and the solitary bee surrogate LC_50_ = grey dashed vertical line) are represented by vertical lines on the EED. Exceedance of these endpoints is calculated by subtracting the cumulative probability from one. Grey horizontal lines represent individual soil samples below the analytical limit of detection, and black dots indicate samples for which insecticide residues could be quantified. The red line indicates the line of best fit to the data via maximum likelihood estimation (MLE) with associated 95% confidence intervals (pink shading).


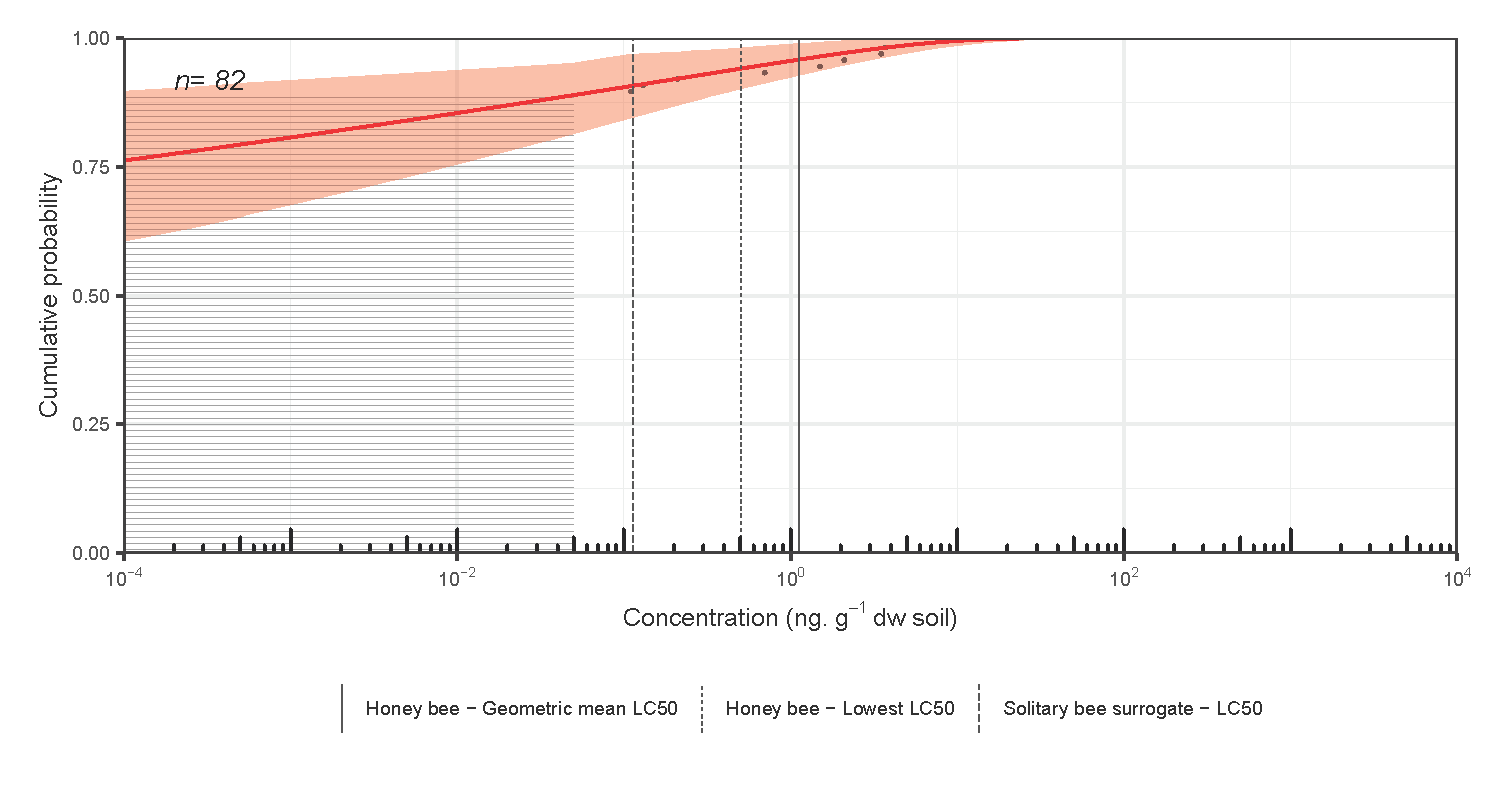


**Table S1**. Calculations of the volume, and therefore, mass of soil excavated by a female hoary squash bee (*Peponapis pruinosa*) to construct an underground nest, from which to determine potential insecticide exposure via soil. Nest dimensions follow data from Mathewson (1968)^2^.

| **Part of Nest** | **Soil volume/ mass** |
| --- | --- |
| a. Main vertical shaft  (7 mm diameter x 18 cm long) | 6.92 cm^3^ |
| b. Antechamber  (7 mm diameter x 6 cm long) | 2.31 cm^3^ |
| c. 5 brood cells  (7 mm diameter x 7.3 cm long = 2.81 cm^3^ each) | 14.04 cm^3^ |
| d. 5 brood caps  (7 mm diameter x 1 cm thick = 0.3847 cm^3^ each)  Estimated from our own observations | 1.92 cm^3^ |
| Total volume of soil excavated  (a + b + c + d) | 25.19 cm^3^ |
| Total mass of soil excavated  (Total volume x Bulk density (BD) of loam; BD = 1.33 g/cm^3^) | **33.51 g** |

**Table S2**. Summary of pesticide exposure routes, exposure types, exposure amount, and exposure period for the hoary squash bee (*Peponapis pruinosa*).

| **Exposure Route** | **Developmental Stage** | **Exposure type** | **Exposure amount** | **Period of Exposure** |
| --- | --- | --- | --- | --- |
| Soil | Adult Female | Contact | 33.51 g ^2*^ | Construction of 1 nest  ~30 days |
| Soil | Larva | Contact | Putatively not exposed ^2^ | 10 months |
| Nectar | Adult Female | Oral | <<780 mg; based on pollen-foraging honey bee ^3^ | 30+ days |
| Nectar | Adult Male | Oral | Unknown, <adult female | 30+ days |
| Nectar | Larva | Oral | Unknown, < adult females | 15 days |
| Pollen | Larva | Oral | 54.2 mg ^4,*^ | 15 days |
| Pollen | Larva | Contact | Unknown | 15 days |
| Pollen | Adult Female | Contact | 246 mg (5x larval exposure) ^4,*^ | 30 days |
| Pollen | Adult Female | Oral | Unknown | During oocyte maturation^5^ |

* = present study

**Table S3**. All residues detected in soil, pollen, and nectar of *Cucurbita*-crop growing systems in Ontario, Canada, 2016, showing limits of detection and quantification (LOD/LOQ), maximum concentrations, geometric mean concentrations, and frequency of detection for each residue (n = 29 samples, nd = not detected, dnq = detected, not quantifiable). Frequency of detection is based on samples in which residues were detectable but not necessarily quantifiable (>LOD).

|  |  | **Soil** | | | | **Pollen** | | | | **Nectar** | | | |
| --- | --- | --- | --- | --- | --- | --- | --- | --- | --- | --- | --- | --- | --- |
|  |  | **LOD/ LOQ** | **Max Conc.** | **Mean Conc.** | **Freq. Detection** | **LOD/ LOQ** | **Max Conc.** | **Mean Conc.** | **Freq. Detection** | **LOD/**  **LOQ** | **Max Conc.** | **Mean Conc.** | **Freq. Detection** |
| **Type of pesticide** | **Active ingredient** | ppb | ng a.i./g matrix | ng a.i./g matrix | % | ppb | ng a.i./g matrix | ng a.i./g matrix | % | ppb | ng a.i./g matrix | ng a.i./g matrix | % |
| **Insecticide** | Clothianidin | 1/4 | 5.9 | 2.0 | 34 | 2/8 | nd | nd | 0 | 1/4 | nd | nd | 0 |
|  | Imidacloprid | 3/9 | 41.6 | 3.0 | 21 | 2/8 | 4.3 | 4.3 | 3 | 1/4 | 1.1 | 0.9 | 3 |
|  | Thiamethoxam | 5/20 | dnq | - | 3 | 2/8 | nd | nd | 0 | 0.6/2 | nd | nd | 0 |
|  | Chlorantraniliprole | 6/20 | 148.5 | 36.8 | 24 | 3/10 | 68 | 68 | 3 | 2/6 | nd | nd | 0 |
|  | Carbaryl | 1/4 | 352.8 | 14.2 | 10 | 2/7 | 31.1 | 16.5 | 7 | 0.6/2 | nd | nd | 0 |
|  | Methomyl | 7/20 | nd | - | 0 | 2/6 | nd | nd | 0 | 0.3/0.9 | 0.5 | 0.4 | 7 |
|  | Dimethoate | 2/6 | nd | - | 0 | 0.5/2 | 6.2 | 6.2 | 3 | 0.4/1 | 0.5 | 0.5 | 3 |
|  |  |  |  |  |  |  |  |  |  |  |  |  |  |
| **Fungicide** | Pyraclostrobin | 4/10 | 16.7 | 3.8 | 10 | 1/4 | 399.6 | 29.6 | 7 | 0.4/1 | 2 | 2 | 3 |
|  | Picoxystrobin | 0.4/1 | nd | nd | 0 | 0.7/2 | 110.2 | 4.6 | 10 | 0.2/0.7 | 0.3 | 0.3 | 3 |
|  | Boscalid | 3/9 | 374.9 | 46.2 | 31 | 2/6 | 25 | 17.8 | 7 | 2/8 | nd | nd | 0 |
|  | Propamocarb | 20/50 | 64.4 | 23.0 | 10 | 0.9/3 | 402.6 | 222.1 | 14 | 0.2/0.5 | 74.5 | 11.2 | 17 |
|  | Quinoxyfen | 5/10 | 14.7 | 7.9 | 3 | 3/8 | 94.9 | 79.1 | 7 | 1/3 | nd | nd | 0 |
|  | Difenoconazole | 4/10 | 40.7 | 18.9 | 14 | 2/5 | 22.4 | 16.5 | 7 | 0.7/2 | nd | nd | 0 |
|  |  |  |  |  |  |  |  |  |  |  |  |  |  |
| **Herbicide** | Napropamide | 1/4 | 59.4 | 2.8 | 7 | 0.4/1 | 5.0 | 5.0 | 3 | 0.1/0.4 | nd | nd | 0 |
|  | Linuron | 5/10 | 0.8 | 0.8 | 0 | 1/4 | nd | nd | 0 | 0.8/2 | nd | nd | 0 |

**Table S4**. Physical, chemical, and environmental fate properties of imidacloprid, clothianidin, thiamethoxam and chlorantraniliprole^4-14^

| **Active Ingredient** | **Imidacloprid** | **Clothianidin** | **Thiamethoxam** | **Chlorantraniliprole** |
| --- | --- | --- | --- | --- |
| IUPAC name | (*NE*)-*N*-[1-[(6-chloropyridin-3-yl)methyl]imidazolidin-2-ylidene]nitramide^6^ | 1-[(2-chloro-1,3-thiazol-5-yl)methyl]-3-methyl-2-nitroguanidine^9^ | (*NE*)-*N*-[3-[(2-chloro-1,3-thiazol-5-yl) methyl]-5-methyl-1,3,5-oxadiazinin-4-ylidene]nitramide^13^ | 5-bromo-*N*-[4-chloro-2-methyl-6-(methylcarbamoyl)phenyl]-2-(3-chloropyridin-2-yl)pyrazole-3-carboxamide^16^ |
| Insecticide class | Neonicotinoid^6^ | Neonicotinoid^9^ | Neonicotinoid^13^ | Anthranilic diamide^16^ |
| Formula | C_9_H_10_ClN_5_O_2_^6^ | C_6_H_8_ClN_5_O_2_S^9^ | ‎C_8_H_10_ClN_5_O_3_S^13^ | C_18_H_14_BrCl_2_N_5_O_2_^16^ |
| CAS No. | 138261-41-3^6^ | 210880-92-5^9^ | 153719-23-4^13^ | 500008-45-7^16^ |
| Molecular Weight (g/mol) | 255.662^6^ | 249.673^9^ | 291.71^13^ | 483.147^16^ |
| Mode of action | nicotinic acetylcholine receptor agonist^6^ | nicotinic acetylcholine receptor agonist^9^ | nicotinic acetylcholine receptor agonist^13^ | ryanodine receptor activator^16^ |
| Solubility in water | 6.1X10 ^2^ mg/L at 20°C^6^ | 327 mg/L at 20°C^9,11^ | 4.1X10^3^ mg/L at 25°C^13^ | 0.9-1.0 mg/L at 20°C, pH 7^16,17^ |
| Adsorption to Particles (soil) K_oc_ | 156-800^7^ | 60^10^ | 56.2-68.4^14,15^ | 153-526^16,18^ |
| Volatility (air)  Vapour pressure | 7X10^-12^ mmHg at 25°C^6^ | 9.8X10^-13^ mmHg at 25°C^9^ | 4.95X10^-11^ mmHg at 25°C^13,14^ | 1.2X10^-14^ mmHg at 25°C^16^ |
| Log K_ow_ | 0.57 at 21°C^6^ | 0.7 at 25°C^9^ | -0.13 at 25°C^13,14^ | 2.76^16^ |
| Bio-degradation DT_50_ (days) | 69-997^8^ | 277-1386^12^ | 7-72^15^ | 123-561^17^ |

K_oc_ = organic carbon-water partition coefficient; K_ow_ = n-Octanol/Water Partition Coefficient; DT_50_ = Disappearance Time 50

**Table S5**. Exposure endpoints for amounts of soil handled by the hoary squash bee in acute (48h, 2.23 g soil) and chronic (30 days, 33.5 g soil) exposure scenarios based on various effect endpoints in the scientific literature^8-16^. HB = honey bee, SB = solitary bee.

| **Insecticide** | **Exposure type** | **Effect endpoint** | **Effect endpoint concentration**  **(ng a.i./bee)**  **A** | **Exposure amount (g soil/bee)**  **B** | **Exposure endpoint**  **(ng a.i./g soil)**  **(A/B)** | **Source for effect endpoint** |
| --- | --- | --- | --- | --- | --- | --- |
| **Clothianidin** | Acute | Geomean HB LD_50_ | 35.88 | 2.23 | 15.16 | 19-21 |
|  |  | Lowest HB LD_50_ | 22 | 2.23 | 9.29 | 20 |
|  |  | SB Surrogate LD_50_ | 3.588 | 2.23 | 1.52 | 22 |
|  | Chronic | Geomean HB LD_50_ | 35.88 | 33.5 | 1.01 | 19-21 |
|  |  | Lowest HB LD_50_ | 22 | 33.5 | 0.62 | 20 |
|  |  | SB Surrogate LD_50_ | 3.588 | 33.5 | 0.10 | 22 |
| **Imidacloprid** | Acute | Geomean HB LD_50_ | 40.03 | 2.23 | 16.91 | 19-23 |
|  |  | Lowest HB LD_50_ | 18 | 2.23 | 7.61 | 20 |
|  |  | SB Surrogate LD_50_ | 4.003 | 2.23 | 1.69 | 22 |
|  | Chronic | Geomean HB LD_50_ | 40.03 | 33.5 | 1.13 | 19-23 |
|  |  | Lowest HB LD_50_ | 18 | 33.5 | 0.51 | 20 |
|  |  | SB Surrogate LD_50_ | 4.003 | 33.5 | 0.113 | 22 |
| **Thiamethoxam** | Acute | Geomean HB LD_50_ | 25.64 | 2.23 | 10.83 | 19,21,23,24 |
|  |  | Lowest HB LD_50_ | 24 | 2.23 | 10.14 | 19 |
|  |  | SB Surrogate LD_50_ | 2.564 | 2.23 | 1.08 | 22 |
|  | Chronic | Geomean HB LD_50_ | 25.64 | 33.5 | 0.722 | 19,21,23,24 |
|  |  | Lowest HB LD_50_ | 24 | 33.5 | 0.676 | 19 |
|  |  | SB Surrogate LD_50_ | 2.564 | 33.5 | 0.072 | 22 |
| **Chlorantraniliprole** | Acute | Lowest HB LD_50_ | >87,500 | 2.23 | 36547.09 | 25 |
|  |  | SB Surrogate LD_50_ | >8750 | 2.23 | 3654.71 | 22,25 |
|  | Chronic | Lowest HB LD_50_ | >87,500 | 33.5 | 2432.84 | 25 |
|  |  | SB Surrogate LD_50_ | >8750 | 33.5 | 243.28 | 22,25 |

**Table S6**. Exceedance probabilities (i.e. the frequency that effect endpoints were exceeded) for 100% translocation of residues from soil to bee, with upper and lower limits of the 95% confidence interval and the exposure concentrations associated with each effect endpoint, for all systemic insecticides detected in soil of *Cucurbita* and other agricultural field crops in Ontario for both chronic (30 days, 33.5 g soil) and acute (48h, 2.23 g soil) exposure scenarios. HB = honey bee, SB = solitary bee. Data for field crop soils from MOECC data^1^.

| **Insecticide** | **Exposure type** | **Crop system** | **Effect endpoint** | **% Exceedance** | **Lower limit of 95% CI** | **Upper limit of 95% CI** | **Effect concentration**  **ng ai/g soil** |
| --- | --- | --- | --- | --- | --- | --- | --- |
| Clothianidin | Chronic | *Cucurbita* | Lowest HB LC_50_ | 44.3 | 65.9 | 24.5 | 0.6 |
| Clothianidin | Chronic | *Cucurbita* | Geomean LC_50_ | 35.8 | 54.9 | 17.7 | 1.0 |
| Clothianidin | Chronic | *Cucurbita* | SB Surrogate LD_50_ | 68.7 | 89.1 | 41.5 | 0.1 |
| Clothianidin | Acute | *Cucurbita* | Lowest HB LC_50_ | 2.4 | 4.6 | 0.4 | 9.3 |
| Clothianidin | Acute | *Cucurbita* | Geomean LC_50_ | 0.5 | 1.4 | 0.04 | 15.2 |
| Clothianidin | Acute | *Cucurbita* | SB Surrogate LD_50_ | 28.3 | 44.5 | 14.2 | 1.5 |
| Imidacloprid | Chronic | *Cucurbita* | Lowest HB LC_50_ | 57.8 | 100 | 26.2 | 0.5 |
| Imidacloprid | Chronic | *Cucurbita* | Geomean LC_50_ | 39.8 | 99.9 | 17.2 | 1.1 |
| Imidacloprid | Chronic | *Cucurbita* | SB Surrogate LD_50_ | 85.4 | 100 | 45.4 | 0.1 |
| Imidacloprid | Acute | *Cucurbita* | Lowest HB LC_50_ | 8.9 | 17.0 | 0.0 | 7.6 |
| Imidacloprid | Acute | *Cucurbita* | Geomean LC_50_ | 3.5 | 9.0 | 0.0 | 16.9 |
| Imidacloprid | Acute | *Cucurbita* | SB Surrogate LD_50_ | 31.2 | 95.6 | 13.7 | 1.7 |
| Chlorantraniliprole | Chronic | *Cucurbita* | Lowest HB LC_50_ | 0.0 | 0.0 | 0.0 | 2432.8 |
| Chlorantraniliprole | Chronic | *Cucurbita* | SB Surrogate LD_50_ | 1.6 | 3.4 | 0.12 | 243.3 |
| Chlorantraniliprole | Acute | *Cucurbita* | Lowest HB LC_50_ | 0.0 | 0 | 0 | 36547.1 |
| Chlorantraniliprole | Acute | *Cucurbita* | SB Surrogate LD_50_ | 0.0 | 0.0 | 0 | 3654.7 |
| Clothianidin | Chronic | Field crops | Lowest HB LC_50_ | 92.4 | 97.0 | 86.0 | 0.6 |
| Clothianidin | Chronic | Field crops | Geomean LC_50_ | 87.7 | 94.0 | 80.1 | 1.0 |
| Clothianidin | Chronic | Field crops | SB Surrogate LD_50_ | 98.8 | 99.8 | 96.5 | 0.1 |
| Clothianidin | Acute | Field crops | Lowest HB LC_50_ | 27.3 | 35.3 | 19.1 | 9.3 |
| Clothianidin | Acute | Field crops | Geomean LC_50_ | 11.7 | 17.6 | 5.8 | 15.2 |
| Clothianidin | Acute | Field crops | SB Surrogate LD_50_ | 81.9 | 89.7 | 73.6 | 1.5 |
| Imidacloprid | Chronic | Field crops | Lowest HB LC_50_ | 5.9 | 9.7 | 2.1 | 0.5 |
| Imidacloprid | Chronic | Field crops | Geomean LC_50_ | 4.2 | 7.1 | 1.3 | 1.1 |
| Imidacloprid | Chronic | Field crops | SB Surrogate LD_50_ | 9.2 | 15.3 | 3.9 | 0.1 |
| **Insecticide** | **Exposure type** | **Crop system** | **Effect endpoint** | **% Exceedance** | **Lower limit of 95% CI** | **Upper limit of 95% CI** | **Effect concentration**  **ng ai/g soil** |
| Imidacloprid | Acute | Field crops | Lowest HB LC_50_ | 0.8 | 1.8 | 0.0 | 7.6 |
| Imidacloprid | Acute | Field crops | Geomean LC_50_ | 0.2 | 0.7 | 0.0 | 16.9 |
| Imidacloprid | Acute | Field crops | SB Surrogate LD_50_ | 3.3 | 5.6 | 0.9 | 1.7 |
| Thiamethoxam | Chronic | Field crops | Lowest HB LC_50_ | 37.4 | 45.8 | 29.4 | 0.7 |
| Thiamethoxam | Chronic | Field crops | Geomean LC_50_ | 35.7 | 44.0 | 27.8 | 0.7 |
| Thiamethoxam | Chronic | Field crops | SB Surrogate LD_50_ | 78.4 | 85.8 | 69.7 | 0.1 |
| Thiamethoxam | Acute | Field crops | Lowest HB LC_50_ | 0.0 | 0.3 | 0.0 | 10.1 |
| Thiamethoxam | Acute | Field crops | Geomean LC_50_ | 0.0 | 0.2 | 0.0 | 10.8 |
| Thiamethoxam | Acute | Field crops | SB Surrogate LD_50_ | 25.6 | 33.1 | 17.8 | 1.1 |

**Table S7.** Parameters (shape and rate) and associated 95% confidence interval for the gamma (clothianidin and chlorantraniliprole in *Cucurbita* crops; all residues in field crops) or log-normal (imidacloprid and thiamethoxam in *Cucurbita* crops) model that was fit to the distribution of measured concentrations of each insecticide and the Akaike Information Criterion (AIC) for each model.

| **Insecticide** | **Sample depth/ cm** | **Crop type** | **shape** | **shape_2.5** | **shape_97.5** | **rate** | **rate_2.5** | **rate_97.5** | **AIC** |
| --- | --- | --- | --- | --- | --- | --- | --- | --- | --- |
| Clothianidin | 0-15 | Cucurbita | 0.34304 | 0.14723 | 0.66789 | 0.22847 | 0.13871 | 0.39616 | 76.905 |
| Imidacloprid | 0-15 | Cucurbita | -0.05037 | -2.51720 | 0.91093 | 1.58923 | 0.21634 | 3.15571 | 56.837 |
| Chlorantraniliprole | 0-15 | Cucurbita | 0.09636 | 0.03414 | 0.20079 | 0.00521 | 0.00285 | 0.01329 | 107.633 |
| Clothianidin | 0-15 | Field crops | 1.06754 | 0.75564 | 1.53303 | 0.15031 | 0.09975 | 0.23856 | 508.432 |
| Imidacloprid | 0-15 | Field crops | 0.02543 | 0.01082 | 0.04471 | 0.10976 | 0.04719 | 0.39991 | 91.718 |
| Thiamethoxam | 0-15 | Field crops | 0.53550 | 0.39736 | 0.73792 | 0.64797 | 0.43633 | 1.09491 | 205.267 |

**Table S8.** Exceedance of 3 effect endpoints (honey bee geometric mean LD_50_, honey bee lowest LD_50_, solitary bee surrogate LD_50_) for less than 100% translocation (75%, 50%, 25%, 10%) of neonicotinoid insecticide (clothianidin, imidacloprid, thiamethoxam) residue from soil to female ground-nesting bees as they construct nests in either an acute (48 h, 2.23 g soil) or chronic (30 days, 33.5 g soil) exposure scenario for two crop types (*Cucurbita* crops, field crops) based on exposure for the hoary squash bee (*Peponapis pruinosa*). Data for field crop soils from MOECC data^1^

| **Crop type** | **Compound** | **Exposure Scenario** | **Effect Endpoint** | | **Trans-location**  **(%)** | | **Exceedance (%)** | | **Lower 95% CI** | | **Upper 95% CI** | | **Effect Concentration (ng ai/g soil)** | |
| --- | --- | --- | --- | --- | --- | --- | --- | --- | --- | --- | --- | --- | --- | --- |
| *Cucurbita* | Clothianidin | Acute | Honey bee Geometric mean LC_50_ | | 10% | | 0 | | 0 | | 0 | | 151.6052 | |
|  |  |  |  | | 25% | | 0 | | 0 | | 0 | | 60.6421 | |
|  |  |  |  | | 50% | | 0.01 | | 0.1 | | 0 | | 30.3210 | |
|  |  |  |  | | 75% | | 0.15 | | 0.52 | | 0.01 | | 20.2140 | |
|  |  |  | Honey bee Lowest LC_50_ | | 10% | | 0 | | 0 | | 0 | | 92.9577 | |
|  |  |  |  | | 25% | | 0 | | 0.03 | | 0 | | 37.1831 | |
|  |  |  |  | | 50% | | 0.22 | | 0.71 | | 0.01 | | 18.5915 | |
|  |  |  |  | | 75% | | 1.07 | | 2.39 | | 0.12 | | 12.3944 | |
|  |  |  | Solitary bee Surrogate LC_50_ | | 10% | | 0.53 | | 1.35 | | 0.04 | | 15.1607 | |
|  |  |  |  | | 25% | | 6.08 | | 10.14 | | 1.80 | | 6.0643 | |
|  |  |  |  | | 50% | | 15.88 | | 25.77 | | 7.21 | | 3.0321 | |
|  |  |  |  | | 75% | | 23.01 | | 36.46 | | 11.10 | | 2.0214 | |
|  |  | Chronic | Honey bee Geometric mean LC_50_ | | 10% | | 1.96 | | 3.85 | | 0.31 | | 10.1070 | |
|  |  |  |  | | 25% | | 11.33 | | 18.8 | | 4.45 | | 4.0428 | |
|  |  |  |  | | 50% | | 23.01 | | 36.46 | | 11.1 | | 2.0214 | |
|  |  |  |  | | 75% | | 30.48 | | 47.55 | | 15.21 | | 1.3476 | |
|  |  |  | Honey bee Lowest LC_50_ | | 10% | | 5.84 | | 9.75 | | 1.72 | | 6.1972 | |
|  |  |  |  | | 25% | | 19.35 | | 31.18 | | 9.19 | | 2.4789 | |
|  |  |  |  | | 50% | | 32.03 | | 49.68 | | 15.92 | | 1.2394 | |
| **Table S8 continued** | | | | | | | | | | | | | | |
| **Crop type** | **Compound** | **Exposure Scenario** | **Effect Endpoint** | | **Trans-location**  **(%)** | | **Exceedance (%)** | | **Lower 95% CI** | | **Upper 95% CI** | | **Effect Concentration (ng ai/g soil)** | |
| *Cucurbita* | Clothianidin | Chronic |  | | 75% | | 39.37 | | 59.67 | | 19.62 | | 0.8263 | |
|  |  |  | Solitary bee Surrogate LC_50_ | | 10% | | 35.78 | | 54.9 | | 17.68 | | 1.0107 | |
|  |  |  |  | | 25% | | 51.13 | | 73.42 | | 29.67 | | 0.4043 | |
|  |  |  |  | | 50% | | 60.79 | | 82.91 | | 35.58 | | 0.2021 | |
|  |  |  |  | | 75% | | 65.60 | | 86.87 | | 39.11 | | 0.1348 | |
|  |  |  |  |  | |  | |  | |  | |  | |  |
| *Cucurbita* | Imidacloprid | Acute | Honey bee Geometric mean LC_50_ | 10% | | 0.09 | | 1.19 | | 0 | | 169.1370 | |  |
|  |  |  |  | 25% | | 0.47 | | 2.80 | | 0 | | 67.6548 | |  |
|  |  |  |  | 50% | | 1.38 | | 4.99 | | 0 | | 33.8274 | |  |
|  |  |  |  | 75% | | 2.43 | | 7.00 | | 0 | | 22.5516 | |  |
|  |  |  | Honey bee Lowest LC_50_ | 10% | | 0.38 | | 2.51 | | 0 | | 76.0563 | |  |
|  |  |  |  | 25% | | 1.61 | | 5.50 | | 0 | | 30.4225 | |  |
|  |  |  |  | 50% | | 4.04 | | 9.82 | | 0 | | 15.2113 | |  |
|  |  |  |  | 75% | | 6.49 | | 13.38 | | 0 | | 10.1408 | |  |
|  |  |  | Solitary bee Surrogate LC_50_ | 10% | | 3.54 | | 8.96 | | 0 | | 16.9137 | |  |
|  |  |  |  | 25% | | 9.96 | | 18.47 | | 0 | | 6.7655 | |  |
|  |  |  |  | 50% | | 18.74 | | 32.09 | | 3.37 | | 3.3827 | |  |
|  |  |  |  | 75% | | 25.59 | | 66.38 | | 12.06 | | 2.2552 | |  |
|  |  | Chronic | Honey bee Geometric mean LC_50_ | 10% | | 5.76 | | 12.43 | | 0 | | 11.2758 | |  |
|  |  |  |  | 25% | | 14.64 | | 25.79 | | 0.18 | | 4.5103 | |  |
|  |  |  |  | 50% | | 25.59 | | 66.38 | | 12.06 | | 2.2552 | |  |
|  |  |  |  | 75% | | 33.57 | | 98.77 | | 14.41 | | 1.5034 | |  |
|  |  |  | Honey bee Lowest LC_50_ | 10% | | 13.17 | | 23.72 | | 0.03 | | 5.0704 | |  |
|  |  |  |  | 25% | | 27.58 | | 81.16 | | 12.64 | | 2.02817 | |  |
|  |  |  |  | 50% | | 42.10 | | 100 | | 18.75 | | 1.0141 | |  |
| Table S8 continued | | | | | | | | | | | | | |  |
| **Crop type** | **Compound** | **Exposure Scenario** | **Effect Endpoint** | **Trans-location**  **(%)** | | **Exceedance (%)** | | **Lower 95% CI** | | **Upper 95% CI** | | **Effect Concentration (ng ai/g soil)** | |  |
| *Cucurbita* | Imidacloprid | Chronic | Honey bee Lowest LC_50_ | 75% | | 51.29 | | 100 | | 23.08 | | 0.6761 | |  |
|  |  |  | Solitary bee Surrogate LC_50_ | 10% | | 39.76 | | 99.98 | | 17.24 | | 1.1276 | |  |
|  |  |  |  | 25% | | 60.38 | | 100 | | 27.55 | | 0.4510 | |  |
|  |  |  |  | 50% | | 74.52 | | 100 | | 36.09 | | 0.2255 | |  |
|  |  |  |  | 75% | | 81.36 | | 100 | | 41.47 | | 0.1503 | |  |
| Field crops | Clothianidin | Acute | Honey bee Geometric mean LC_50_ | | 10% | | 0 | | 0 | | 0 | | 151.6052 | |
|  |  |  |  | | 25% | | 0.02 | | 0.14 | | 0 | | 60.6421 | |
|  |  |  |  | | 50% | | 1.31 | | 3.33 | | 0.21 | | 30.3210 | |
|  |  |  |  | | 75% | | 5.65 | | 10.03 | | 1.91 | | 20.2140 | |
|  |  |  | Honey bee Lowest LC_50_ | | 10% | | 0 | | 0 | | 0 | | 92.9577 | |
|  |  |  |  | | 25% | | 0.48 | | 1.62 | | 0.04 | | 37.1831 | |
|  |  |  |  | | 50% | | 7.14 | | 12.04 | | 2.74 | | 18.5915 | |
|  |  |  |  | | 75% | | 17.47 | | 24.31 | | 10.26 | | 12.3944 | |
|  |  |  | Solitary bee Surrogate LC_50_ | | 10% | | 11.72 | | 17.73 | | 5.64 | | 15.1607 | |
|  |  |  |  | | 25% | | 43.23 | | 51.81 | | 34.93 | | 6.0643 | |
|  |  |  |  | | 50% | | 66.33 | | 75.23 | | 57.83 | | 3.0321 | |
|  |  |  |  | | 75% | | 76.35 | | 84.49 | | 67.76 | | 2.0214 | |
|  | Clothianidin | Chronic | Honey bee Geometric mean LC_50_ | | 10% | | 24.26 | | 31.72 | | 16.49 | | 10.1070 | |
|  |  |  |  | | 25% | | 57.56 | | 66.27 | | 48.77 | | 4.0428 | |
|  |  |  |  | | 50% | | 76.35 | | 84.49 | | 67.76 | | 2.0214 | |
|  |  |  |  | | 75% | | 83.76 | | 90.73 | | 75.71 | | 1.3476 | |
|  |  |  | Honey bee Lowest LC_50_ | | 10% | | 42.42 | | 50.97 | | 34.05 | | 6.1972 | |
|  |  |  |  | | 25% | | 71.65 | | 80.37 | | 63.01 | | 2.4789 | |
|  |  |  |  | | 50% | | 85.00 | | 91.72 | | 77.12 | | 1.2394 | |
|  |  |  |  | | 75% | | 89.89 | | 95.21 | | 82.99 | | 0.8263 | |
| Table S8 Continued | | | | | | | | | | | | | | |
| **Crop type** | **Compound** | **Exposure Scenario** | **Effect Endpoint** | | **Trans-location**  **(%)** | | **Exceedance (%)** | | **Lower 95% CI** | | **Upper 95% CI** | | **Effect Concentration (ng ai/g soil)** | |
| Field crops | Clothianidin | Chronic | Solitary bee Surrogate LC_50_ | | 10% | | 87.68 | | 93.67 | | 80.2 | | 1.0107 | |
|  |  |  |  | | 25% | | 95.06 | | 98.30 | | 90.03 | | 0.4043 | |
|  |  |  |  | | 50% | | 97.57 | | 99.40 | | 93.98 | | 0.2021 | |
|  |  |  |  | | 75% | | 98.40 | | 99.68 | | 95.54 | | 0.1348 | |
| Field crops | Imidacloprid | Acute | Honey bee Geometric mean LC_50_ | | 10% | | 0 | | 0 | | 0 | | 169.1370 | |
|  |  |  |  | | 25% | | 0 | | 0 | | 0 | | 67.6548 | |
|  |  |  |  | | 50% | | 0.02 | | 0.19 | | 0 | | 33.8274 | |
|  |  |  |  | | 75% | | 0.08 | | 0.43 | | 0 | | 22.5516 | |
|  |  |  | Honey bee Lowest LC_50_ | | 10% | | 0 | | 0 | | 0 | | 76.0563 | |
|  |  |  |  | | 25% | | 0.03 | | 0.24 | | 0 | | 30.4225 | |
|  |  |  |  | | 50% | | 0.23 | | 0.81 | | 0 | | 15.2113 | |
|  |  |  |  | | 75% | | 0.51 | | 1.39 | | 0 | | 10.1408 | |
|  |  |  | Solitary bee Surrogate LC_50_ | | 10% | | 0.18 | | 0.70 | | 0 | | 16.9137 | |
|  |  |  |  | | 25% | | 0.94 | | 2.14 | | 0.03 | | 6.7655 | |
|  |  |  |  | | 50% | | 1.97 | | 3.85 | | 0.24 | | 3.3827 | |
|  |  |  |  | | 75% | | 2.75 | | 5.01 | | 0.47 | | 2.2552 | |
|  |  | Chronic | Honey bee Geometric mean LC_50_ | | 10% | | 0.43 | | 1.24 | | 0 | | 11.2758 | |
|  |  |  |  | | 25% | | 1.53 | | 3.16 | | 0.13 | | 4.5103 | |
|  |  |  |  | | 50% | | 2.75 | | 5.01 | | 0.47 | | 2.2552 | |
|  |  |  |  | | 75% | | 3.56 | | 6.25 | | 0.84 | | 1.5034 | |
|  |  |  | Honey bee Lowest LC_50_ | | 10% | | 1.33 | | 2.83 | | 0.09 | | 5.0704 | |
|  |  |  |  | | 25% | | 2.94 | | 5.28 | | 0.54 | | 2.0282 | |
|  |  |  |  | | 50% | | 4.37 | | 7.50 | | 1.16 | | 1.0141 | |
|  |  |  |  | | 75% | | 5.25 | | 8.81 | | 1.58 | | 0.6761 | |
|  |  |  |  | |  | |  | |  | |  | |  | |
| Table S8 Continued | | | | | | | | | | | | | | |
| **Crop type** | **Compound** | **Exposure Scenario** | **Effect Endpoint** | | **Trans-location**  **(%)** | | **Exceedance (%)** | | **Lower 95% CI** | | **Upper 95% CI** | | **Effect Concentration (ng ai/g soil)** | |
| Field crops | Imidacloprid | Chronic | Solitary bee Surrogate LC_50_ | | 10% | | 4.16 | | 7.19 | | 1.07 | | 1.1276 | |
|  |  |  |  | | 25% | | 6.14 | | 10.16 | | 2.00 | | 0.4510 | |
|  |  |  |  | | 50% | | 7.69 | | 12.70 | | 2.65 | | 0.2255 | |
|  |  |  |  | | 75% | | 8.60 | | 14.26 | | 2.95 | | 0.1503 | |
| Field crops | Thiamethoxam | Acute | Honey bee Geometric mean LC_50_ | | 10% | | 0 | | 0 | | 0 | | 108.3263 | |
|  |  |  |  | | 25% | | 0 | | 0 | | 0 | | 43.3305 | |
|  |  |  |  | | 50% | | 0 | | 0 | | 0 | | 21.6653 | |
|  |  |  |  | | 75% | | 0 | | 0.04 | | 0 | | 14.4435 | |
|  |  |  | Honey bee Lowest LC_50_ | | 10% | | 0 | | 0 | | 0 | | 101.4084 | |
|  |  |  |  | | 25% | | 0 | | 0 | | 0 | | 40.5634 | |
|  |  |  |  | | 50% | | 0 | | 0 | | 0 | | 20.2817 | |
|  |  |  |  | | 75% | | 0 | | 0.05 | | 0 | | 13.5211 | |
|  |  |  | Solitary bee Surrogate LC_50_ | | 10% | | 0.02 | | 0.18 | | 0 | | 10.8326 | |
|  |  |  |  | | 25% | | 2.02 | | 4.65 | | 0.4 | | 4.3331 | |
|  |  |  |  | | 50% | | 10.37 | | 15.91 | | 4.87 | | 2.1665 | |
|  |  |  |  | | 75% | | 18.71 | | 25.68 | | 11.15 | | 1.4444 | |
|  |  | Chronic | Honey bee Geometric mean LC_50_ | | 10% | | 0.26 | | 1.07 | | 0.02 | | 7.2218 | |
|  |  |  |  | | 25% | | 5.93 | | 10.26 | | 2.12 | | 2.8887 | |
|  |  |  |  | | 50% | | 18.71 | | 25.68 | | 11.15 | | 1.4444 | |
|  |  |  |  | | 75% | | 28.48 | | 36.27 | | 20.02 | | 0.9629 | |
|  |  |  | Honey bee Lowest LC_50_ | | 10% | | 0.36 | | 1.37 | | 0.03 | | 6.7606 | |
|  |  |  |  | | 25% | | 6.83 | | 11.45 | | 2.63 | | 2.7042 | |
|  |  |  |  | | 50% | | 20.20 | | 27.31 | | 12.46 | | 1.3521 | |
|  |  |  |  | | 75% | | 30.14 | | 38.01 | | 21.58 | | 0.9014 | |
|  |  |  |  | |  | |  | |  | |  | |  | |
| Table S8 Continued | | | | | | | | | | | | | | |
| **Crop type** | **Compound** | **Exposure Scenario** | **Effect Endpoint** | | **Trans-location**  **(%)** | | **Exceedance (%)** | | **Lower 95% CI** | | **Upper 95% CI** | | **Effect Concentration (ng ai/g soil)** | |
| Field crops | Thiamethoxam | Chronic | Solitary bee Surrogate LC_50_ | | 10% | | 35.70 | | 43.76 | | 27.11 | | 0.7222 | |
|  |  |  |  | | 25% | | 56.88 | | 65.8 | | 48.07 | | 0.2889 | |
|  |  |  |  | | 50% | | 69.26 | | 77.86 | | 60.10 | | 0.1444 | |
|  |  |  |  | | 75% | | 74.98 | | 83.11 | | 66.36 | | 0.0963 | |

Table S9. Hazard quotients (HQ) for each active ingredient, and pesticide type (insecticide, fungicide) in three exposure matrices (soil, pollen, nectar) and both developmental stages (adult female or larvae) based on honey bee LD_50_ values/10 to represent solitary bee endpoints, and mean residue concentration in the exposure routes for all pesticide residues found detected on 18 *Cucurbita-*crop farms in Ontario in 2016. Numbers shown in bold face are combined hazard quotients for a pesticide type, an exposure matrix, or a developmental stage. Where “ND” is indicated, residues were not detected, where “NQ” is indicated, residues were not quantifiable in samples. a.i. = active ingredient. Honey bee LD_50_ values obtained from the literature as indicated.^19-21,23-27^

|  | **Honey bee LC_50_ Endpoint** | | **SOIL** | | **POLLEN** | | | | | **NECTAR** | | **COMBINED HAZARD QUOTIENT** | | | | |
| --- | --- | --- | --- | --- | --- | --- | --- | --- | --- | --- | --- | --- | --- | --- | --- | --- |
|  | **Contact** | **Oral** | **Mean Conc.** | **HQ (SB)** | **Mean Conc.** | **HQ (SB)** | | **HQ (SB)** | | **Mean Conc. in Matrix** | **HQ (SB)** | **∑HQ (SB)** | | | | |
|  |  |  | **in Matrix** | **Adult Female** | **in Matrix** | **Larvae** | | **Adult Female** | |  | **Adult Female** |  |  |  |  |  |
|  |  |  |  | **(Contact)** |  | **(Oral)** | | **(Contact)** | |  | **(Oral)** |  |  |  |  |  |
|  | ng a.i./bee | ng a.i./bee or ppb | ng a.i./g | =33.5 g/bee *Matrix Conc. /0.1*LD**_50_** | ng a.i./g | =0.0542 g/bee *Matrix Conc. /0.1*LD**_50_** | | =5*0.0542 g/bee* Matrix Conc. /0.1*LD**_50_** | | ng a.i./g | =0.78 g/bee* Matrix Conc. /0.1*LD**_50_** | **Across Exposure Matrix, All Stages** | **Adult Female** | | | **Larvae** |
|  |  |  |  |  |  |  |  |  |  |  |  |  |  |  |  |  |
|  |  |  |  |  |  |  | |  | |  |  |  | |  |  | |
| **INSECTICIDE** ∑HQ |  |  |  | **43.8** |  | **0.6** | | **0.3** | |  | **4.5** | **49.2** | | **48.6** | **0.7** | |
| **Neonicotinoid** ∑HQ |  |  |  | **43.2** |  | **0.6** | | **0.3** | |  | **1.8** | **45.9** | | **45.3** | **0.6** | |
|  |  |  |  |  |  |  | |  | |  |  |  | |  |  | |
| Clothianidin (geomean)^19-21^ | 35.88 | - | 1.95 | 18.2 | ND | 0 | | 0 | | ND | 0 | 18.2 | | 18.2 | 0 | |
| Imidacloprid (geomean)^19,21,23,26,27^ | 40.03 | 3.9 | 2.99 | 25 | 4.3 | 0.6 | | 0.3 | | 0.88 | 1.8 | 27.7 | | 27.1 | 0.6 | |
| Thiamethoxam (geomean)^19,21,24^ | 25.64 | - | NQ | 0 | ND | 0 | | 0 | | ND | 0 | 0 | | 0 | 0 | |
| Chlorantraniliprole^25^ | >81500 | >117800 | 36.82 | 0.2 | 68 | 0.000169 | | 0.00226 | | ND | 0 | 0.202429 | | 0.20226 | 0.000169 | |
| **Table S8 continued** | | | | | | | | | | | | | | | | |
|  | **Honey bee LC_50_ Endpoint** | | **SOIL** | | **POLLEN** | | | | | **NECTAR** | | **COMBINED HAZARD QUOTIENT** | | | | |
|  | **Contact** | **Oral** | **Mean Conc.**  **in Matrix** | **HQ (SB)**  **Adult Female** | **Mean Conc. in Matrix** | | **HQ (SB)**  **Larvae** | | **HQ (SB)**  **Adult Female** | **Mean Conc. in Matrix** | **HQ (SB)**  **Adult Female** | **∑HQ (SB)** | | | | |
|  | **ng a.i./bee** | **ng a.i./bee or ppb** | **ng a.i./g** | **=33.5 g/bee *Matrix Conc. /0.1*LD_50_** | **ng a.i./g** | | **=0.0542 g/bee *Matrix Conc. /0.1*LD_50_** | | **=5*0.0542 g/bee* Matrix Conc. /0.1*LD_50_** | **ng a.i./g** | **=0.78 g/bee* Matrix Conc. /0.1*LD_50_** | **Across Exposure Matrix, All Stages** | **Adult Female** | | | **Larvae** |
| Carbaryl^19^ | 11200 | - | 14.2 | 0.4 | 16.47 | - | | 0.0039 | | ND | 0 | 0.4039 | | 0.4039 | - | |
|  |  |  |  |  |  |  | |  | |  |  |  | |  |  | |
|  |  |  |  |  |  |  | |  | |  |  |  | |  |  | |
| Dimethoate^19^ | - | 56 | ND | 0 | 6.2 | 0.06 | | 0 | | 0.5 | 0.07 | 0.13 | | 0.07 | 0.06 | |
| Methomyl (ppb)^19^ | - | 1.18 | ND | 0 | ND | - | | 0 | | 0.39 | 2.6 | 2.6 | | 2.6 | - | |
|  |  |  |  |  |  |  | |  | |  |  |  | |  |  | |
|  |  |  |  |  |  |  | |  | |  |  |  | |  |  | |
| **FUNGICIDE** ∑HQ |  |  |  | **0.3** |  | **0.00124** | | **0.00618** | |  | **9.20E-04** | **0.30834** | | **0.3071** | **0.00124** | |
|  |  |  |  |  |  |  | |  | |  |  |  | |  |  | |
| Pyraclostrobin^19^ | 100000 | - | 3.8 | 0.00127 | 29.65 | - | | 0.000804 | | 2 | - | **-** | | **-** | - | |
| Picoxystrobin^19^ | 200000 | - | ND | 0 | 4.55 | - | | 0.0000617 | | 0.3 | - | **-** | | **-** | - | |
| Boscalid^19^ | 200000 | 166000 | 46.22 | 0.0774 | 17.82 | 0.0000528 | | 0.000241 | | ND | 0 | 0.0776938 | | 0.077641 | 0.0000528 | |
| Propamocarb^19^ | 100000 | 116000 | 23.03 | 0.0772 | 222.06 | 0.0104 | | 0.00602 | | 11.18 | 7.52E-04 | 0.094372 | | 0.083972 | 0.0104 | |
| Quinoxyfen^19^ | 100000 | 1000000 | 7.86 | 0.0263 | 79.14 | 0.000429 | | 0.00214 | | ND | 0 | 0.028869 | | 0.02844 | 0.000429 | |
| Difenoconazole^19^ | 100000 | 177000 | 18.87 | 0.0632 | 16.46 | 0.000504 | | 0.000446 | | ND | 0 | 0.06415 | | 0.063646 | 0.000504 | |

**References for Supplementary Information**

1. MOECC. Soil neonicotinoid monitoring study (2016). <https://www.ontario.ca/data/soil-neonicotinoid-monitoring-study> (accessed on 9 July 2019)
2. Mathewson, J. A. Nest construction and life history of the eastern cucurbit bee, *Peponapis pruinosa* (Hymenoptera: Apoidea). *Journal of the Kansas Entomological Society* **41**, 255-261 (1968).
3. Rortais, A., Arnold, G., Halm, M. P. & Touffet-Briens, F. Modes of honeybees exposure to systemic insecticides: estimated amounts of contaminated pollen and nectar consumed by different categories of bees. *Apidologie* **36**, 71-83 (2005). doi:10.1051/apido:2004071
4. Willis, D. S. The pollination system of *Cucurbita pepo* and *Peponapis pruinosa* in southern Ontario. MSc. dissertation, University of Guelph, Ontario, Canada (1991).
5. Michener, C. D. The Bees of the World, 2^nd^ ed. 30-32 (The John Hopkins University Press, 2007).
6. National Center for Biotechnology Information. PubChem Database. Imidacloprid, CID=86287518, <https://pubchem.ncbi.nlm.nih.gov/compound/86287518> (accessed on 9 July 2019)
7. TOXNET Toxicology Data Network. Imidacloprid, CASRN: 138261-41-3, <https://toxnet.nlm.nih.gov/cgi-bin/sis/search/a?dbs+hsdb:@term+@DOCNO+7373> (accessed on 9 July 2019)
8. National Pesticide Information Center. Imidacloprid technical factsheet, <http://npic.orst.edu/factsheets/archive/imidacloprid.html> (accessed on 9 July 2019)
9. National Center for Biotechnology Information. PubChem Database. Clothianidin, CID=86287519, <https://pubchem.ncbi.nlm.nih.gov/compound/Clothianidin> (accessed on 9 July 2019)
10. TOXNET Toxicology Data Network. Clothianidin, CASRN: 210880-92-5, <https://toxnet.nlm.nih.gov/cgi-bin/sis/search/a?dbs+hsdb:@term+@DOCNO+7281> (accessed on 9 July 2019)
11. USEPA/Office of Pesticide Programs; Pesticide Fact Sheet-Clothianidin (2003). <https://www3.epa.gov/pesticides/chem_search/reg_actions/registration/fs_PC-044309_30-May-03.pdf> (accessed on 9 July 2019)
12. IUPAC Pesticide Properties Database: Clothianidin, <https://sitem.herts.ac.uk/aeru/iupac/Reports/171.htm> (accessed on 9 July 2019)
13. National Center for Biotechnology Information. PubChem Database. Actara, CID=5821911, <https://pubchem.ncbi.nlm.nih.gov/compound/5821911> (accessed on 9 July 2019)
14. TOXNET Toxicology Data Network. Thiamethoxam, CASRN: 153719-23-4, <https://toxnet.nlm.nih.gov/cgi-bin/sis/search/a?dbs+hsdb:@term+@DOCNO+7938> (accessed on 9 July 2019)
15. IUPAC Pesticide Properties Database: Thiamethoxam, <https://sitem.herts.ac.uk/aeru/iupac/Reports/631.htm> (accessed on 9 July 2019)
16. National Center for Biotechnology Information. PubChem Database. Chlorantraniliprole, CID=11271640, <https://pubchem.ncbi.nlm.nih.gov/compound/Chlorantraniliprole> (accessed on 9 July 2019)
17. IUPAC Pesticide Properties Database: Chlorantraniliprole, <https://sitem.herts.ac.uk/aeru/iupac/Reports/1138.htm> (accessed on 9 July 2019)
18. USEPA/Office of Pesticide Programs; Pesticide Fact Sheet-Chlorantraniliprole (2008). <https://www3.epa.gov/pesticides/chem_search/reg_actions/registration/fs_PC-090100_01-Apr-08.pdf> (accessed on 9 July 2019)
19. US-EPA OPP Pesticide Ecotoxicity Database. United States Environmental Protection Agency, Ecological Fates and Effects Division, Office of Pesticide Programs, Washington DC. Available from, <https://ecotox.ipmcenters.org/> (2019).
20. Iwasa, T., Motoyama, N., Ambrose, J. T. & Roe, R. M. Mechanism for the differential toxicity of neonicotinoid insecticides in the honey bee, *Apis mellifera*. *Crop Protection* **23**, 371–378 (2004). doi:10.1016/j.cropro.2003.08.018
21. Sanchez-Bayo, F. & Goka, K. Pesticide residues and bees – a risk assessment. *PLoS One* **9**, e94482 (2014). doi:10.1371/journal.pone.0094482
22. Arena, M. & Sgolastra, F. Meta-analysis comparing the sensitivity of bees to pesticides. *Ecotoxicology* **23**, 324–334 (2014). doi:10.1007/s10646-014-1190-1
23. Stark, J. D., Jepson, P. C. & Mayer, D. F. Limitation to use of topical toxicity data for prediction of pesticide side effect in the field. *Journal of Economic Entomology* **88**, 1081-1088 (1995). doi:10.1093/jee/88.5.1081
24. EFSA. Conclusion the peer review of the pesticide risk assessment for bees for the active substance thiamethoxam. *EFSA Journal* **11**, 3067 (2013). doi:10.2903/j.efsa.2013.3067
25. Dinter, A., Brugger, K. E., Frost, N-M. & Woodward, M. D. Chlorantraniliprole (Rynaxypyr): a novel DuPont insecticide with low toxicity and low risk for honey bees (*Apis mellifera*) and bumble bees (*Bombus terrestris*) providing excellent tools for uses in integrated pest management. *Julius-Kühn-Archives* **423**, 84-96 (2009).
26. Ruzhong, G., Rui, C. & Liangyan, C. Evaluation on toxicity and safety of imidacloprid to environmental organisms. *Pesticide Science and Administration* **20**(3)(1999).
27. Suchail, S., Guez, D. & Belzunces, L. P. Discrepancy between acute and chronic toxicity induced by imidacloprid and its metabolites in *Apis mellifera*. *Environmental Toxicology and Chemistry* **20**, 2482–2486 (2001). doi:10.1002/etc.5620201113
